# High Glucose Reduces Influenza and Parainfluenza Virus Productivity by Altering Glycolytic Pattern in A549 Cells

**DOI:** 10.3390/ijms26072975

**Published:** 2025-03-25

**Authors:** Kareem Awad, Maha Abdelhadi, Ahmed M. Awad

**Affiliations:** 1 Institute of Biomedicine, Faculty of Medicine, University of Turku, 20520 Turku, Finland; 2 Institute of Pharmaceutical and Drug Industries Research, National Research Centre, Giza 12622, Egypt; 3 Medical Faculty, Ruprecht-Karls-University of Heidelberg, 69117 Heidelberg, Germany; 4 Academy of Scientific Research & Technology (ASRT-STARS), Cairo 11516, Egypt; 5 Institute of Medical Research and Clinical Studies, National Research Center, Giza 12622, Egypt; mahahadi39@yahoo.com; 6 Research and Innovation Office, California State University Channel Islands, Camarillo, CA 93012, USA; ahmed.awad@csuci.edu

**Keywords:** influenza, Sendai virus, glycolysis, phosphofructokinase, TGF-β1

## Abstract

Influenza A virus is responsible for annual epidemics and occasional pandemics leading to significant mortality and morbidity in human populations. Parainfluenza viruses also contribute to lung infections and chronic lung disease. In this study, we investigated the effect of high glucose on the productivity of influenza A and Sendai (murine parainfluenza type 1) viruses in A549 immortalized cells. A glycolytic pattern of infection was determined by monitoring the release of lactate and phosphofructokinase (PFK) activity in infected and uninfected cells. qRT-PCR was used to analyze the expression of viral and cellular cytokine mRNA levels in cultured cells. The data show that the productivity of both influenza and Sendai viruses was reduced in A549 cells cultured in high-glucose conditions. This was accompanied by increased lactate production and altered PFK activity profile. Endogenous or virus infection-induced interferon β (IFN-β) mRNA expression was significantly decreased in high glucose compared to normal glucose status during early times of infection. Unlike in Sendai virus-infected cells, H1N1 virus reversed the significant increase in transforming growth factor β1 (TGF-β1) mRNA expression due to increased glucose concentration during early infection times. In conclusion, high glucose may have a negative effect on influenza and parainfluenza productivity in vitro. This effect may be considered when evaluating personalized therapeutic/diagnostic markers in infection-accompanied hyperglycemic status.

## 1. Introduction

Influenza A virus records annually significant worldwide mortality and morbidity in human populations [1]. Parainfluenza virus infections are associated with the development of chronic lung diseases due to their protein and RNA persistence in lung innate immune cells [2]. The primary targets of influenza and parainfluenza virus replication are the lung epithelial cells and blood mononuclear cells, resulting in destruction of infected cells, which may lead to lung failure [1,2,3].

Metabolic reprogramming is a hallmark of viral-infected cells because virus replication, propagation, and assembly into new virions require energy; thus, infection dynamics can be altered if there are changes in host cell metabolism [4,5,6]. Experimental data from different influenza variants of different productivities and infection dynamics in different cell types show differential and specific time course metabolic patterns [6,7,8]. Influenza virus strains have demonstrated distinct glycolytic patterns, which include increases in the expression of rate limiting enzymes in glycolysis and increases in lactate formation [8,9].

The human lung epithelial cell line A549 has been widely used to investigate responses to influenza A and Sendai virus infections [3,10]. Sendai virus (a murine parainfluenza type 1 virus) is a widely used model for human parainfluenza viruses. The extracellular matrix environment is important in disorders and development while targeting cellular processes and pathways and remains a promising antiviral strategy shown to be effective during influenza virus infection [11,12]. The airways cells release interferons (IFNs) as a main defense mechanism to overcome viral infection, and any deficiencies in IFN inductions may lead to severe progression of viral infectivity [13]. Detailed information on the kinetics of either IFN or IFN-induced protein production in human lung A549 cells in response to influenza A virus infection have been reported [2,3,10]. Transforming growth factor β1 (TGF-β1) is a multifunctional cytokine that acts differentially through multiple mechanisms either to enhance or suppress cell proliferation based on the environmental cytokine profile and the status of the affected cell type [14,15,16].

It is now widely evident that different cell types tolerate specific borders of normal glucose concentration before changing their behavior due to hyperglycemia or abnormal glucose concentration in their growth environment [16,17,18]. Therefore, the purpose of the present study was to investigate the effect of high glucose on the infectivity of A549 cells with influenza and Sendai viruses in vitro.

## 2. Results

### 2.1. Determination of Influenza and Sendai Virus Replication in Normal, +10 mM, and +25 mM Glucose Concentrations

**Figure 1:**
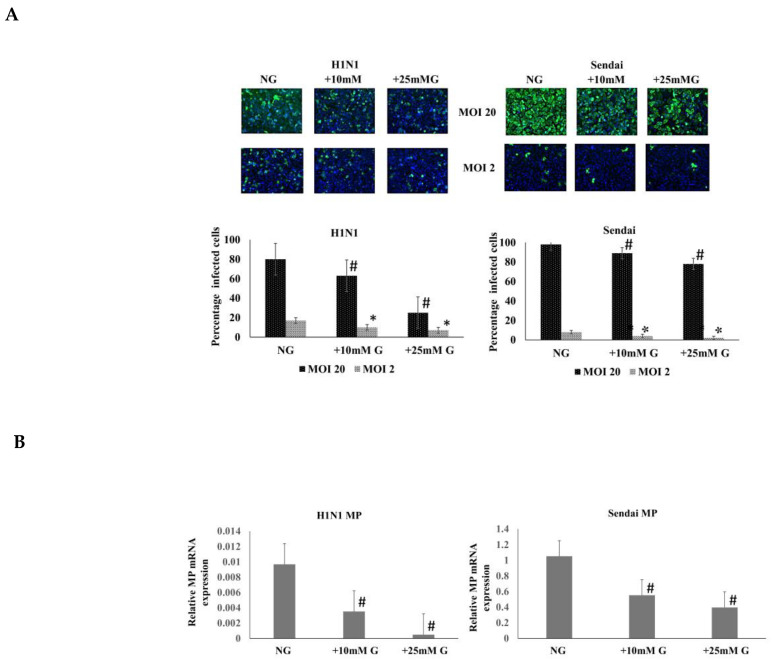
Influenza and Sendai virus replication in normal, +10 mM, and +25 mM glucose (G) concentrations. A549 cells were cultured in DMEM medium supplemented with 10% FCS in normal glucose (NG; 25 mM), +10 mM G (35 mM), and +25 mM G (50 mM) concentrations. Cells remained uninfected or they were infected with IAV H1N1 or Sendai virus (MOI 2 or 20) for 16 h. (**A**) The IF staining of H1N1 or Sendai virus nucleoportein (NP) was carried out in A549 cells control and post infection (PI) in NG, +10 mM G and +25 mM G. H1N1/Sendai virus NP was detected using specific rabbit anti influenza A and Sendai virus NP antibodies. Lower panel indicates the quantitation of the number of virus-infected cells in different virus doses and glucose concentrations. (**B**) A549 cells were infected with H1N1 and Sendai viruses (MOI 20) for 16 h followed by the isolation of total cellular RNA for qRT-PCR analysis. Equal amounts of total cellular RNA were analyzed by H1N1 and Sendai virus matrix (M) gene-specific mRNA primers, for quantitating viral mRNA expression. The results show a significant reduction in IAV and Sendai virus M mRNA expression in higher glucose concentration compared to NG culture. *n* = 4, * or # refers to statistical difference *p* < 0.05 between the samples in the corresponding higher glucose concentrations compared to NG.

In order to analyze whether high glucose concentrations have an effect on virus replication in human lung epithelial cell model A549 cells, we infected the cells with various doses (MOI 2 and 20) of influenza A and Sendai viruses in different glucose concentrations. Cells were fixed at 16 h after infection and processed for immunofluorescence (IF) microscopic analysis of H1N1 type influenza A virus (IAV) or Sendai virus nucleoprotein (NP) expression (Figure 1A). The number of virus-infected cells was significantly reduced in high glucose concentrations. Similarly infected cells (MOI 20) followed by the isolation of total cellular RNA and qRT-PCR of H1N1 and Sendai virus M mRNA show a significant reduction in M mRNA expression, respectively, in higher glucose concentration compared to normal glucose (NG) conditions (*n* = 4, *p* < 0.05, (Figure 1B). The data indicate that H1N1 or Sendai virus replication is dose-dependently reduced with increasing glucose concentration in cell culture media. Noteworthy to mention that this reduction in both viruses replication in primary human macrophages cultured in higher glucose concentrations was also evidenced and subject to further experimental investigation (Figure S1).

### 2.2. Immunofluorescence Staining of H1N1 or Sendai Virus NP in A549 Cells Control and Infected with H1N1 (MOI 20) for 2–48 h PI in NG, +10 mM G, and +25 mM G

**Figure 2:**
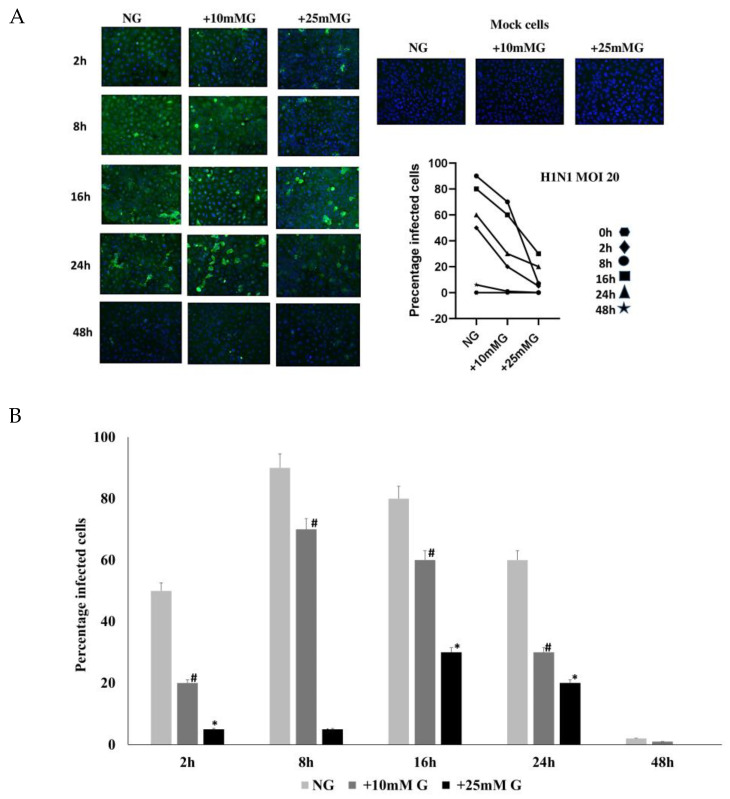
Immunofluorescence staining of H1N1 virus NP in A549 cells control and infected with H1N1 (MOI 20) for 2–48 h post infection (PI) in NG, +10 mM G, and +25 mM G. A549 cells were cultured in DMEM medium supplemented with 10% FCS in normal glucose (25 mM), +10 mM glucose (35 mM), and +25 mM glucose (50 mM). Cells remained uninfected or were infected with H1N1 virus (MOI 20) 2–48 h. H1N1 virus NP was detected using rabbit anti H1N1 NP antibodies. (**A**) The IF results show a significant reduction in NP expression in higher glucose concentrations compared to NG culture. (**B**) Quantification of IF images, *n* = 4, * or # refers to statistical difference *p* < 0.05 between the samples in the corresponding higher glucose concentrations compared to NG.

**Figure 3:**
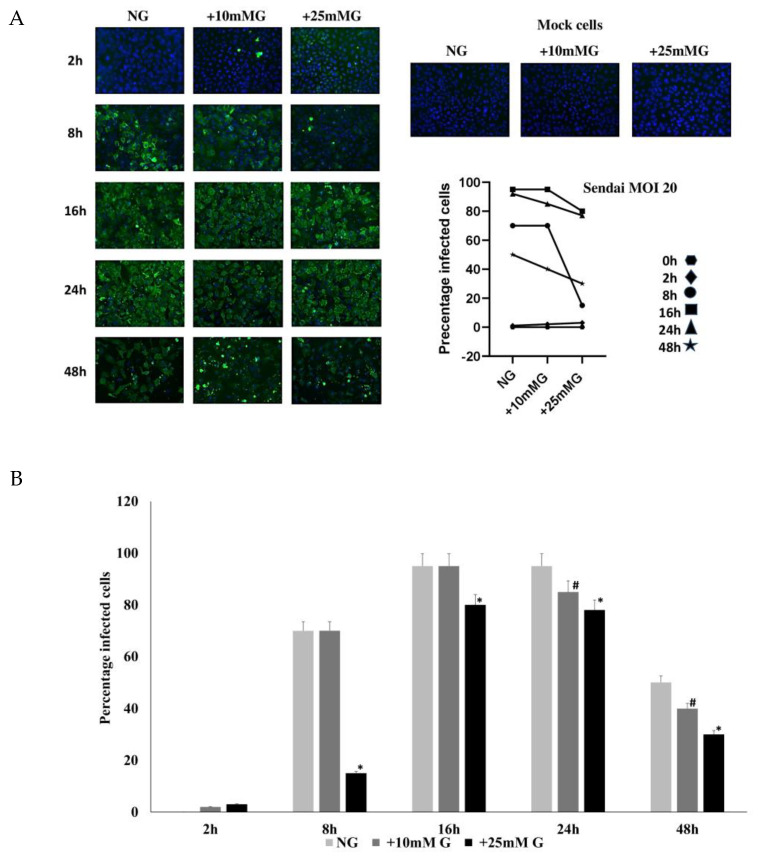
Immunofluorescence staining of Sendai virus NP in A549 cells control and infected with Sendai (MOI 20) for 2–48 h post infection (PI) in NG, +10 mM G, and +25 mM G. A549 cells were cultured in DMEM medium supplemented with 10% FCS in normal glucose (25 mM), +10 mM glucose (35 mM), and +25 mM glucose (50 mM). Cells remained uninfected or were infected with Sendai virus (MOI 20) for 2–48 h. Sendai virus NP was detected using mouse monoclonal anti Sendai NP antibodies. (**A**) The IF results show a significant reduction in NP expression in higher glucose concentration compared to NG culture. (**B**) The quantification of IF images, *n* = 4, * or # refers to statistical difference *p* < 0.05 between the samples in the corresponding higher glucose concentrations compared to NG.

Next, we analyzed the kinetics of viral protein expression in A549 cells infected with IAV H1N1 (Figure 2; MOI 20) or Sendai virus (Figure 3; MOI 20) for different times. Virus-infected cells were fixed at different times (2–48 h) after infection, and the cells were processed for immunofluorescence microscopy. There was a significant reduction in viral NP expression in higher glucose concentrations (+10 mM G and +25 mM G) in cells infected with H1N1 (Figure 2) or Sendai viruses (Figure 3) compared to NG culture conditions at all time points where virus protein expression was detectable.

### 2.3. Lactate Concentration (nmol/µL) in Culture Media of A549 Cells Control and Infected with H1N1 or Sendai Virus (MOI 10) at 2–48 h PI in NG, +10 mM G, and +25 mM G

**Figure 4:**
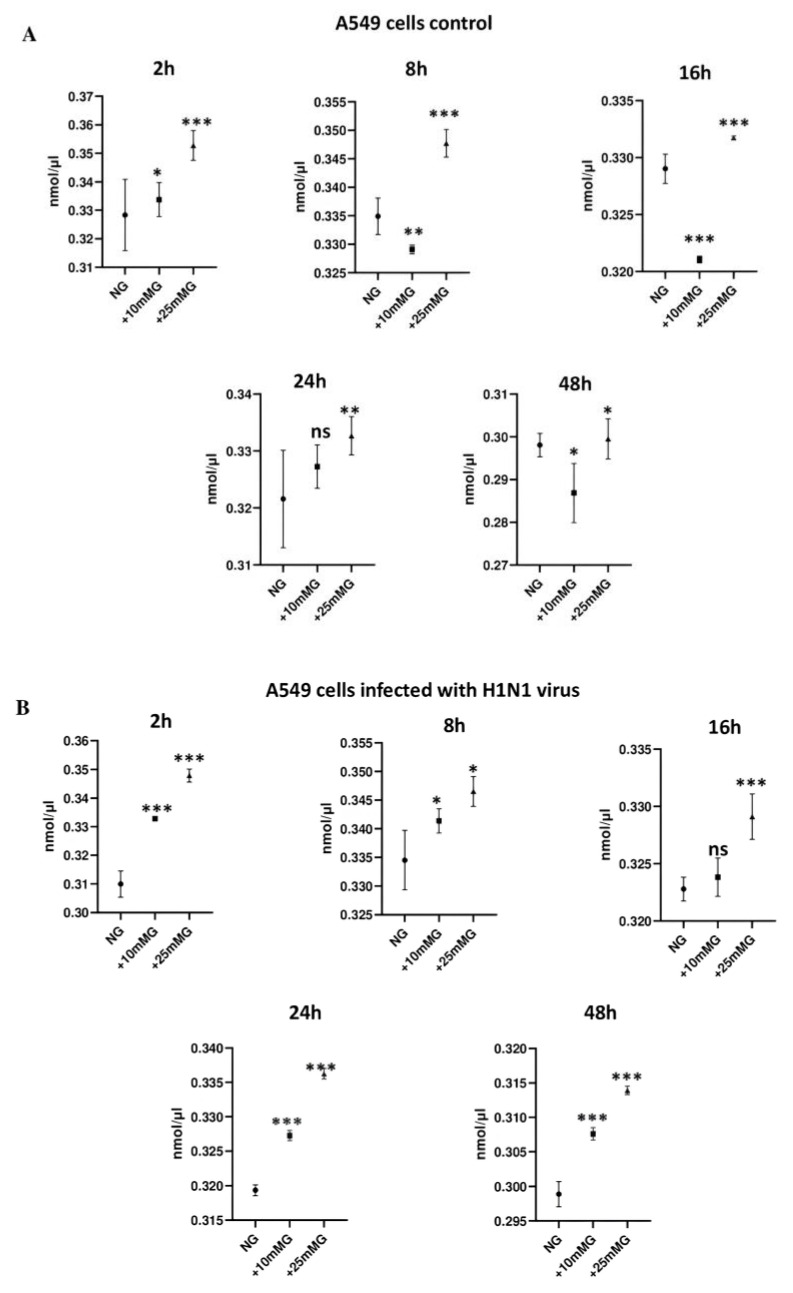

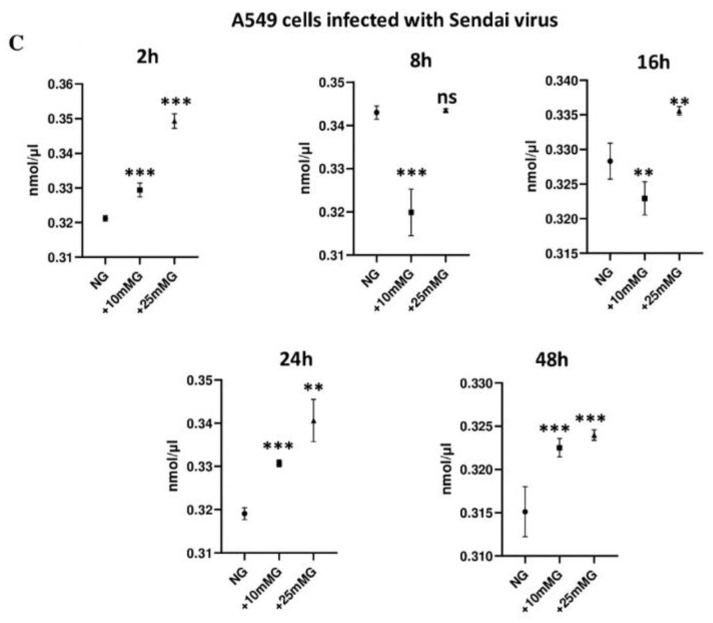
Lactate concentration (nmol/µL) in culture media of A549 cells control and infected with H1N1 or Sendai virus (MOI 10) at 2–48 h post infection (PI) in NG, +10 mM G, and +25 mM G. A549 cells were cultured in DMEM medium supplemented with 10% FCS in normal glucose (25 mM), +10 mM glucose (35 mM), and +25 mM glucose (50 mM). Cells remained uninfected (**A**) or they were infected with H1N1 virus (**B**) or Sendai virus (**C**) for 2, 8, 16, 24, and 48 h. Lactate concentration was measured in the supernatants of cultured cells collected at the different time points of PI, *n* = 4, * refers to statistical difference between the samples in the corresponding higher glucose concentrations compared to NG, *** *p* < 0.0005, ** *p* < 0.005, and * *p* < 0.05.

Next, we analyzed lactate concentrations in cell culture supernatants of virus-infected and uninfected cells in different glucose concentrations. The results show that in uninfected cells (Figure 4A), lactate concentration significantly increased in +25 mM G compared to the control NG group at all time points, and significantly decreased at 8 and 16 h time points after culture in +10 mM G compared to NG control, which suggests that +10 mM G is still biologically normal for the growth of A549 cells. When cells were infected with H1N1 virus (Figure 4B), the lactate concentration in cell culture supernatants significantly increased at both +10 mM and +25 mM at all time points PI. When cells were infected with Sendai virus (Figure 4C), the lactate concentration significantly increased at all time points in +25 mM G and at 2, 24, and 48 h PI compared to the NG control. These results demonstrate that a lactate increase may be attributed to the increase in glucose, while infection or components from the virus may modulate the onset of lactate release relative to glucose concentration. Lactate increase corresponding to glucose increase in culture medium of primary human immune cells has been also previously observed [16], and can be shown fol-lowing macrophages infection with both viruses (Figure S2).

### 2.4. Phosphofructokinase (PFK) Enzyme Activity (nmol/min/µL) in Culture Media of A549 Cells Control and Infected with H1N1 or Sendai Virus (MOI 10) at 2–48 h PI in NG, +10 mM G, and +25 mM G

**Figure 5:**
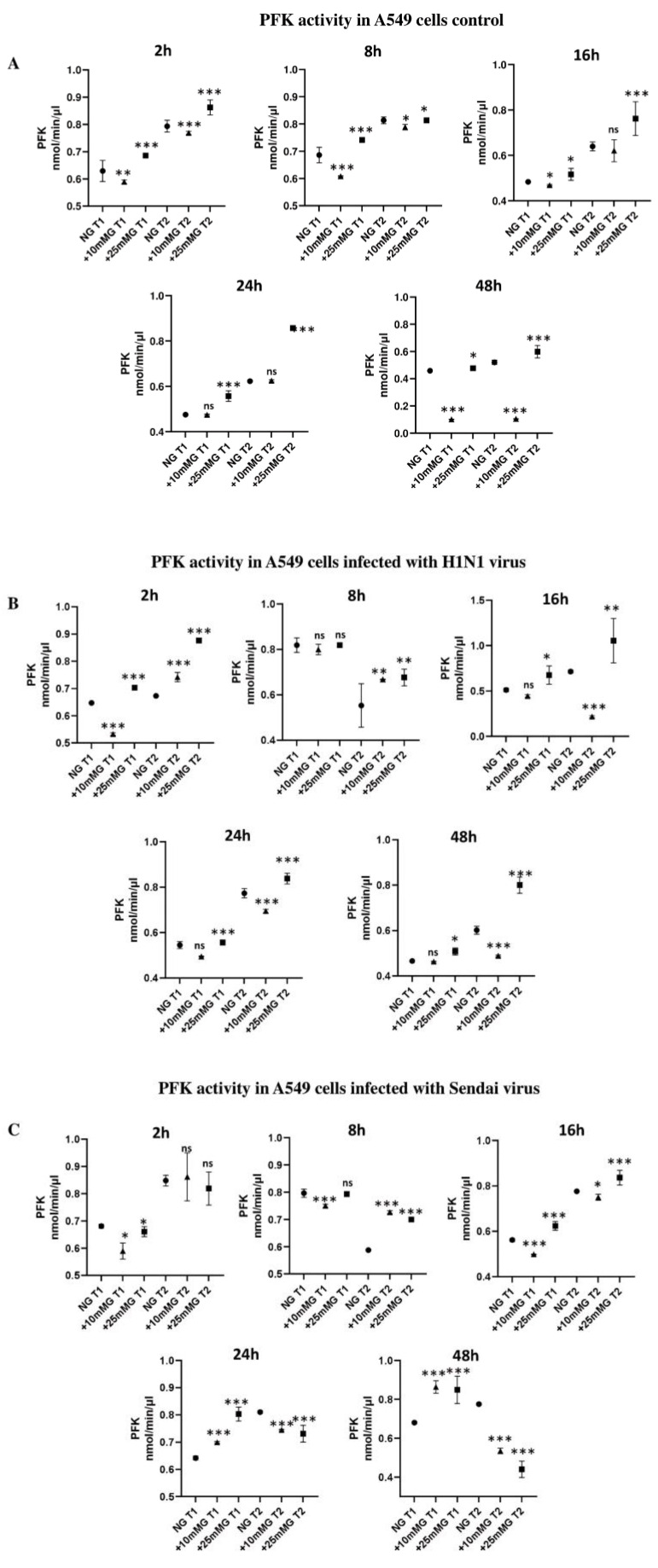
Phosphofructokinase (PFK) enzyme activity (nmol/min/µL) in culture media of A549 control cells and cells infected with H1N1 or Sendai virus (MOI 10) at 2–48 h post infection (PI) in NG, +10 mM G, and +25 mM G. A549 cells were cultured in DMEM medium supplemented with 10% FCS in normal glucose (25 mM), +10 mM glucose (35 mM), and +25 mM glucose (50 mM). Cells remained uninfected (**A**), or they were infected with H1N1 virus (**B**) or Sendai virus (**C**) for 2, 8, 16, 24, and 48 h. PFK activity was measured at two time points kinetic (T1, 5 min and T2, 25 min) in the lysates of cultured cells at the corresponding time point PI, *n* = 4, * refers to statistical difference between the samples in the corresponding higher glucose concentrations compared to NG, *** *p* < 0.0005, ** *p* < 0.005, and * *p* < 0.05.

The results show that in uninfected cells (Figure 5A), the PFK activity profile in both time points of analysis (T1, 5 min and T2, 25 min) significantly decreased after culture in +10 mM G and significantly increased in +25 mM G compared to the control NG group at all time points compared to the NG control. This may indicate that +10 mM G is still biologically normal for the growth of A549 cells. The PFK activity profile changes after infecting A549 cells with either H1N1 or Sendai viruses (Figure 5B,C). Unlike in uninfected cells, when cells were infected with H1N1 virus (Figure 5B), PFK activity at the 8 h time point of PI was higher in the first time point (T1) of measurement and also significantly higher in the second time (T2) point in +10 mM G 2 and 8 h PI compared to the control NG group. Similarly, when cells were infected with Sendai virus (Figure 5C), PFK activity at the 8 h time point of PI was higher in the first time point of measurement, and significantly higher in the second time point in +10 mM G 2 and 8 h PI compared to the control NG group. A significant reduction in PFK activity in the +10 and +25 mM G groups compared to NG group was observed 24 and 48 h PI in the second time point (T2) of measurement. All observations suggest that infection or virus-related components contribute to a reduced glycolytic pattern.

### 2.5. qRT-PCR Expression of IFN-β mRNA in the Lysates of Uninfected or H1N1 or Sendai Virus-Infected A549 Cells in Normal, +10 mM Glucose, or +25 mM Glucose Concentration

**Figure 6:**
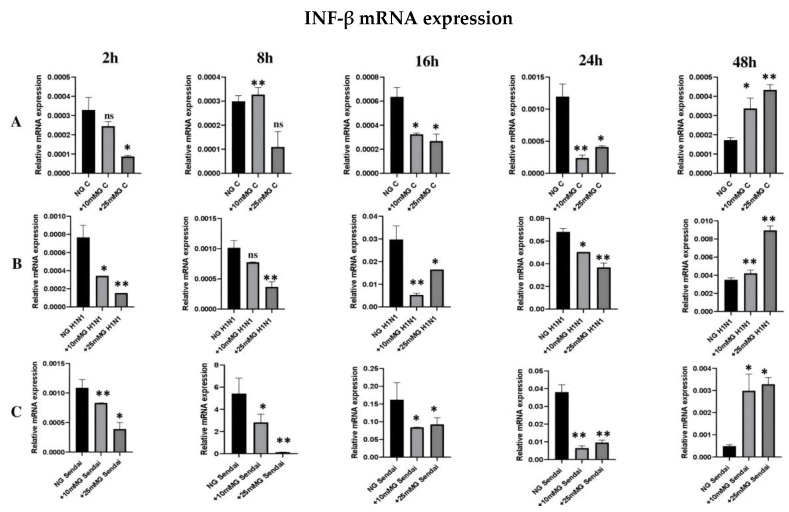
qRT-PCR expression of IFN-β mRNA in total cellular RNA of uninfected or H1N1 or Sendai virus-infected A549 cells in normal, +10 mM glucose, or +25 mM glucose concentration. 10^6^ A549 cells were cultured in DMEM medium supplemented with 10% FCS in normal glucose (25 mM), +10 mM glucose (35 mM), and +25 mM glucose (50 mM). Cells remained uninfected (**A**) or they were infected with H1N1 virus (**B**) or Sendai virus (**C**) for 2, 8, 16, 24, and 48 h. Cells were collected at different times after infection, and total cellular RNA was isolated. cDNA was subjected to qRT-PCR to detect the relative expression of IFNβ (β actin mRNA level was used as a reference), *n* = 3, * refers to statistical difference between the samples in the corresponding higher glucose concentrations compared to NG, * *p* < 0.05, and ** *p* < 0.005. All tests were carried out in duplicate or triplicate.

Next, we analyzed the expression of antiviral IFN-**β** mRNA in virus-infected cells in different glucose concentrations. A reduction in the levels of the IFN-β transcripts was recorded in cDNA from uninfected (endogenous; Figure 6A), H1N1 (Figure 6B), or Sendai (Figure 6C) virus-infected A549 cells in +10 mM G and +25 mM glucose concentration until 24 h of infection compared to the NG status. The level of IFN-β mRNA was significantly increased in +10 mM G and +25 mM G compared to the NG status after 48 h both in infected and uninfected cells. The levels of IFN-β mRNA were 10- to 20-fold higher in H1N1 infected cells compared to uninfected cells after 8–16 h PI. The levels of IFN-β mRNA were 100- to 1000-fold higher in Sendai virus-infected cells compared to uninfected cells after 8–16 h PI. This IFNβ mRNA significant reduction in higher glucose culture demonstrates the clear negative effect of high glucose on the release of specific virus components either considered endogenous or released due to infection.

### 2.6. qRT-PCR Analysis of TGF-β1 mRNA Expression in Total Cellular RNA of Uninfected or H1N1 or Sendai Virus-Infected A549 Cells in Normal, +10 mM Glucose, or +25 mM Glucose Concentration

**Figure 7:**
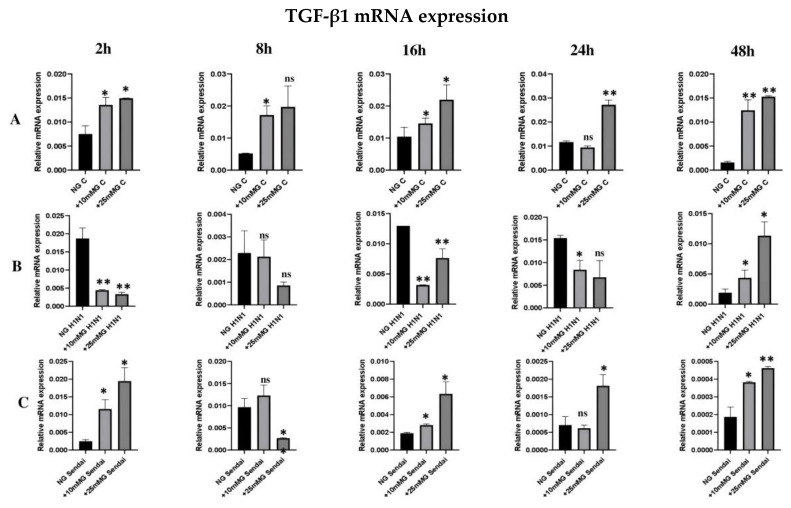
qRT-PCR expression of TGF-β1 mRNA in total cellular RNA of uninfected or H1N1 or Sendai virus-infected A549 cells in normal or +10 mM glucose or +25 mM glucose concentration. 10^6^ A549 cells were cultured in DMEM medium supplemented with 10% FCS in normal glucose (25 mM), +10 mM glucose (35 mM), and +25 mM glucose (50 mM). Cells remained uninfected (**A**), or they were infected with H1N1 virus (**B**) or Sendai virus (**C**) for 2, 8, 16, 24, and 48 h. At different times after infection, cells were collected and total cellular RNA was isolated. cDNA was subjected to qRT-PCR to detect the relative expression of TGF-β1 (β actin mRNA level was used as a reference), *n* = 3, * refers to statistical difference between the samples in the corresponding higher glucose concentrations compared to NG, * *p* < 0.05 and ** *p* < 0.005. All tests were carried out in duplicates or triplicates.

In uninfected A549 cells, TGF-β1 transcript significantly increased in +10 mM G and +25 mM G compared to NG at all time points (Figure 7A). This profile differentially changed upon infection with either H1N1 or Sendai viruses in time points 2–24 h and remained in infected cells 48 h PI (Figure 7B,C). In time points 2–24 h, post H1N1 (unlike Sendai) virus infection, TGF-β1 mRNA levels showed a reduction in +10 mM G and +25 mM G compared to that seen in NG conditions (Figure 7B,C). Although high glucose levels in culture medium lead to a significant increase in TGF-β1 mRNA levels, H1N1 (2–24 h PI) or Sendai (8 h PI) virus infection reverses this during early infection times, suggesting the interaction between virus components and possible regulators of the complex TGF-β1 pathway.

## 3. Discussion

Influenza remains a considerable public health problem. Therefore, it is necessary to develop ways to control this infection by not only targeting virus specific proteins but also targeting the host specific cellular metabolic processes that the virus may utilize for replication and propagation [19,20]. This may provide novel solutions to control or ameliorate the burden of disease caused by emerging influenza virus strains.

The immunofluorescence staining of H1N1 or Sendai virus nucleoproteins in H1N1 or Sendai virus-infected A549 cells at different MOIs (1–20) or different time points (2–48 h) PI in NG, +10 mM G, and +25 mM G demonstrated a significant reduction in viral protein expression in higher glucose concentrations compared to NG culture conditions. Previous studies show some discrepancies clinically and in different cell types when investigating influenza-glucose relationships. H1N1 virus infection directly consumed glucose from cultured U937 immune cells 24 h PI [7]. An older study reported the inhibition of glucose utilization and a decrease in lactate production in homogenates of brains of mice infected with different viruses, including influenza virus, and related it to the function of the virus concentration, temperature, and time of infection [21]. Unlike older evidence, increased glycolysis evidenced by lactate increase can now finger print infection from different viruses, including influenza [8,9]. The present study showed a significant increase in lactate concentration in hyperglycemic culture (+25 mM) in A549 cells compared to normal glucose concentration either before or after infection. We can, however, not see an increase or a decrease in cell culture lactate concentration due to infection, but unlike in uninfected cells, the lactate profile at all time points was higher in +10 mM G than normal glucose post H1N1 infection.

Elevated glucose levels (12 mM) have been associated with endothelial cell inflammation and the destruction of the epithelial junctional complex and increased influenza severity in culture models involving human epithelial cells [22]. Other studies have reported that elevated glucose levels can directly affect the activity of ATPases in epithelial cells during influenza infection [20,23]. Clinically, elevated blood glucose levels have been associated with severe viral infection [24,25]. The discrepancy between different studies may be the result of different disease models, the pathogenesis of different influenza virus strains, infection time points, or the cell metabolic microenvironment [26,27].

In the present study, PFK1 enzyme activity has shown a distinct profile in normal cells at all measured time points that is a significant activity decrease in +10 mM G followed by an increase in +25 mM G compared to normoglycemic cells. This profile changed post H1N1 or Sendai virus infection in such a way that increased PFK activity was seen in +10 mM G 8hPI compared to the normal glucose culture, and a decrease in PFK activity was seen in high glucose medium compared to normal glucose 24–48 h PI. Previously, it has been shown that differential PFK1 activity and gene expression in different cell types upon H1N1 infection is based on the previous metabolic state of the infected cells [6,8]. The infection may thus change the rate of glycolysis and lactate production, which has been observed by other studies as well [8,9,21].

We observed a generalized significant reduction in endogenous, H1N1, or Sendai virus-induced IFN-β mRNA expression in A549 cells in higher glucose culture compared to normal glucose culture (2–24 h PI). This may support the recent studies that related this inhibition to lactate formation [9]. As an alternative explanation, the high cellular glucose concentrations may lead to the reduced expression of viral RNAs, reducing the ability of the infection to stimulate the RIG-I pathway and IFN-**β** gene expression. The increase in IFN-β mRNA expression 48 h PI in high glucose compared to normoglucose culture needs a further mechanistic explanation.

Initially, TGF-β1 was thought to stimulate cell proliferation, but there is accumulating evidence that TGF-β1 is a bifunctional regulator depending on the target cell [14]. We showed that the TGF-β1 transcript in uninfected A549 cells significantly increased in +10 mM G and +25 mM G compared to NG at all time points analyzed. This profile differentially changed upon infection with either H1N1 or Sendai viruses in time points 2–24 h PI. We agree with others who concluded that epithelial-derived TGF-β1 acts as a pro-viral factor influencing early responses during influenza A infection; they demonstrated that TGF-β1 acts to suppress early IFN-β responses, leading to an increased viral burden and pathology in a mouse model [28].

This study highlights certain cellular changes occurring during the H1N1 and Sendai virus infection of A549 cells in different glucose culture conditions and how these changes can affect the replication and production of these viruses. The pattern of glucose metabolism was specific during each studied virus infection at each glucose concentration. One common feature is the significant decrease in IFN-β mRNA expression during the peak of infection (8–24 h PI) in hyperglycemic compared to normal glycemic medium. This was accompanied by a significant increase in lactate in response to hyperglycemic medium.

It has been known for some time that virus replication can be restricted by the suppression of glucose metabolism, and thus recent studies have been conducted related to the reduction in influenza replication to components from the virus itself and the dynamic regulation of the virus polymerase complex whose function is interfered by glycolysis [5,29,30]. Our data support this theory by showing a specific glycolytic pattern following influenza or parainfluenza infection that is distinguished from the pattern seen in uninfected cells. TGF-β1 mRNA behavior demonstrates the importance of the local microenvironment that, together with metabolic reprogramming, may shape host responses to influenza viral infection at least in the context of interferon production.

In conclusion, our study demonstrates a clear connection between high cellular glucose concentration and reduced influenza and Sendai virus, namely, RNA virus replication. The changes in cellular metabolomics also affected the cytokine gene expression profiles either directly due to glycolysis-dependent events or indirectly by reducing viral replication and RNA expression.

## 4. Methods

### 4.1. Cell Culture and Viruses

The propagation of A549 cells and infection with viruses were carried out as previously described [2,3,10]. In summary, the A549 lung adenocarcinoma cell line (ATCC CCL 185) was obtained from American Type Culture Collection (ATCC, Rockville, MD, USA) and maintained in Dulbecco’s Modified Eagle Medium (DMEM) supplemented with 10% heat inactivated fetal calf serum, 2 mM L-glutamine, 0.6 µg/mL penicillin, and 60 µg/mL streptomycin (Integro, Zaandaam, The Netherlands). Influenza A/California/07/09 (H1N1) wild-type virus and Sendai (strain Cantell) virus were propagated in 11-day-old embryonated eggs as we described before [17]. The infectivity titers of the stock viruses in A549 cells were 6 × 10^7^ PFU/mL and 1 × 10^9^ PFU/mL for H1N1 strain and Sendai virus, respectively. Viruses at different levels of multiplicity of infections (MOI 2–20) were allowed to adhere to A549 cells cultured in normal (25 mM), +10 mM (35 mM), and +25 mM (50 mM) glucose medium for 2–48 h, after which cells were fixed and subjected to immunofluorescence microscopy (IF) (see below). Cell culture supernatants and whole-cell lysates were collected at different times and used for RNA, enzymatic, and other analyses or stored at −80 °C.

### 4.2. Determination of Influenza and Sendai Viruses Productivity in Normal, +10 mM, and +25 mM Glucose Concentrations by Immunofluorescence Staining (IF)

A549 cells were cultured in 96 well plates and infected at different MOIs (1–20) of influenza or Sendai virus stocks for 2–48 h. As previously described [31], cells were fixed with 4% paraformaldehyde for 15 min and permeabilized with 0.1% Triton X-100 in PBS for 5 min. Polyclonal rabbit anti-influenza NP or mouse anti-Sendai virus NP-specific antibody at a dilution of 1:1000 was incubated for 1 h, followed by washing the cells three times with 0.5% BSA in PBS as has been described previously [32,33,34,35]. Goat anti-rabbit or anti-mouse IgG was used as a secondary antibody at 1:1000 dilution (Thermo Scientific, Waltham, MA, USA) and, together with DAPI (1:2500 dilution, Thermo Scientific, Waltham, MA, USA), was added and incubated for 1 h. Wells were washed three times with 0.5% BSA in PBS. The number of FFUs was visualized and counted using an EVOS FL Auto Fluorescence Inverted Microscope (Life Technologies, Carlsbad, CA, USA).

### 4.3. Lactate Assay

Lactate was assayed colorimetrically in the supernatant of A549 cells using the lactate assay kit from Abcam (ab65331, Cambridge, UK) according to the manufacturer’s instructions. A549 cells were infected with H1N1 or Sendai viruses at MOI 20 at 2–48 h post infection (PI) in normal, +10 mM, and +25 mM glucose culture conditions. Following 30 min room temperature incubation with the kit reagents, lactate concentration was measured using a multi-well plate reader at λmax 450 nm (VICTOR^®^ Nivo, PerkinElmer, Waltham, MA, USA). Lactate concentration was calculated using a lactate standard curve.

### 4.4. Phosphofructokinase (PFK) Assay

PFK1 activity was assayed colorimetrically in lysates of A549 cells using the 6-phosphofructokinase activity assay kit from Abcam (ab155898, Cambridge, UK) according to the manufacturer’s instructions. A549 cells were infected with H1N1 or Sendai viruses at MOI 20 at 2–48 h PI in normal, +10 mM, and +25 mM glucose culture conditions. Lysates were prepared by homogenizing the cells (10^6^ cells) with the kit-provided lysis buffer and maintenance on ice for 10 min followed by centrifugation (5 min, 4 °C, 12,000 rpm). Supernatants were collected and considered cell lysates. PFK activity was measured in the cell lysates at two time points (kinetic T1 and T2 (5–25 min)) using a multi-well plate reader (VICTOR^®^ Nivo, PerkinElmer, Waltham, MA, USA) at λmax 450 nm. PFK-1 activity was calculated from the NADH standard curve.

### 4.5. Quantitative Real-Time Reverse Transcriptase PCR (qRT-PCR) of H1N1 and Sendai Viruse Matrix Protein Genes (M1 and M, respectively), IFN-β, or TGF-β1 mRNA

Total cellular RNA was extracted from the lysates of A549 cells infected with H1N1 or Sendai viruses at MOI 20 at 2–48 h PI in normal, +10 mM, and +25 mM glu-cose culture using the PureLink® RNA Mini Kit (Invitrogen, Carlsbad, CA, USA) fol-lowing the protocol of the manufacturer. Prepared RNA were quantified using a nano-spectrophotometer (DeNovix, Wilmington, DE, USA), and equal concentrations (100 ng) were reverse transcribed into cDNA using the High Capacity cDNA Reverse Transcription Kit (Thermo Fisher Scientific, Waltham, MA, USA) according to the manufacturer’s protocol. To amplify individual genes, specific primers for influenza A/California/07/09 (H1N1) M1 (F; TTGAGGCTCTCATGGAATGG, R; GAGCGTGAACACAAATCCTA), Sendai virus M (F; CATGGAGTGAGATACCTAGA, R; CAAGTCAGATACGCTCCCTA), IFN-β (F; CGCAGTGACCATCTATGAGA, R; AGGACTGTCTTCAGATGG), TGF-β1 (F; TTATTGAGCACCTTGGGCAC, R; TCTCTGGGCTTGTTTCCTCAC), and β-actin (F; AGATGGCCACGGCTGCT, R; AACCGCTCATTGCCAATGG) were used. The M influenza gene is conserved in all influenza strains and best suits Rt-PCR virus quantification [36]. qRT-PCR was performed using 100 ng sample cDNA and PowerTrack SYBR Green Kit (Thermo Fish-er Scientific, Waltham, MA, USA) according to the manufacturer’s protocol and using a real-time PCR machine (Rotor Gene Q; Qiagen; Hilden, Germany). The amplification protocol was as follows: initial heat activation, 95 °C for 15 min, then 40 cycles; dena-turation, 94 °C for 15 s; annealing, 52 °C for 30 s; and extension, 72 °C for 30 s. The rel-ative expression levels of mRNA of the studied genes were calculated using the 2−ΔΔCt method, normalized to the mRNA of the housekeeping gene β-actin. Viral gene ex-pression data are presented as the M1 RNA molecules relative copy numbers [36].

### 4.6. Statistical Analysis

Data were analyzed using Microsoft Excel 2013 or the GraphPad Prism software version 8 (La Jolla, San Diego, CA, USA). The Shapiro–Wilk test was used to determine the normality of data. Data are presented as mean ± SD. Statistical significance was assessed by ordinary one-way ANOVA followed by Tukey’s range test. Statistical significance was considered for a *p*-value less than 0.05.

## 5. Conclusions

High glucose concentration in A549 lung epithelial cell-cultured medium reduces the infectivity of influenza and parainfluenza viruses in a time-specific metabolic pattern accompanied by high lactate status. This study signifies the importance of metabolic reprogramming upon viral infection in order to evade the infection or to act for the sake of virus infectivity, and thus targeting these metabolic changes could develop new novel therapeutic approaches. It also suggests that developing potentially new virus infection treatment options should comply with a personalized response in terms of time and grade of infection.

## Data Availability

All data are available from the corresponding author upon reasonable request. All data sets are presented as figures and included within the manuscript.

## Supplementary Materials

The following supporting information can be downloaded at: https://www.mdpi.com/article/10.3390/ijms26072975/s1.
