# Supplementary material for: High Glucose Reduces Influenza and Parainfluenza Virus Productivity by Altering Glycolytic Pattern in A549 Cells
Source: Int J Mol Sci. 2025 Mar 25;26(7):2975. doi: 10.3390/ijms26072975 (PMC11989181; doi:10.3390/ijms26072975)
Supplement: Supplementary file 1 [file ijms-26-02975-s001.zip › ijms-3501254-supplementary.pdf]

Supplementary file

High glucose reduces influenza and parainfluenza virus infection by altering glycolytic pattern in A549 cells

Kareem Awad[1,2,3,4,*], Maha Abdelhadi[5], Ahmed M Awad[6]

[1] Institute of Biomedicine, Faculty of Medicine, University of Turku, 20520 Turku, Finland
[2] Institute of Pharmaceutical and Drug Industries Research, National Research Centre, Giza 12622, Egypt
[3] Medical Faculty, Ruprecht-Karls-University of Heidelberg, 69117 Heidelberg, Germany
[4] Academy of Scientific Research & Technology (ASRT-STARS), Cairo 11516, Egypt
[5] Institute of Medical Research and Clinical Studies, National Research Center, Giza 12622, Egypt; mahahadi39@yahoo.com
[6] Research and Innovation Office, California State University Channel Islands, Camarillo, CA 93012, USA; ahmed.awad@csuci.edu
* Correspondence: kareem.awad@web.de or km.awad@nrc.sci.eg

Preliminary results of the effect of high glucose on influenza and parainfluenza virus infection in primary human macrophages

Results

1- Determination of influenza and Sendai virus replication in normal and +25mM glucose concentrations in primary human macrophages by immunofluorescence (IF) staining

IF of H1N1 or Sendai virus nucleoprotein (NP) show significant reduction in NP expression, in higher glucose concentration compared to NG culture in macrophages from 3 different human donors (D1-D3).

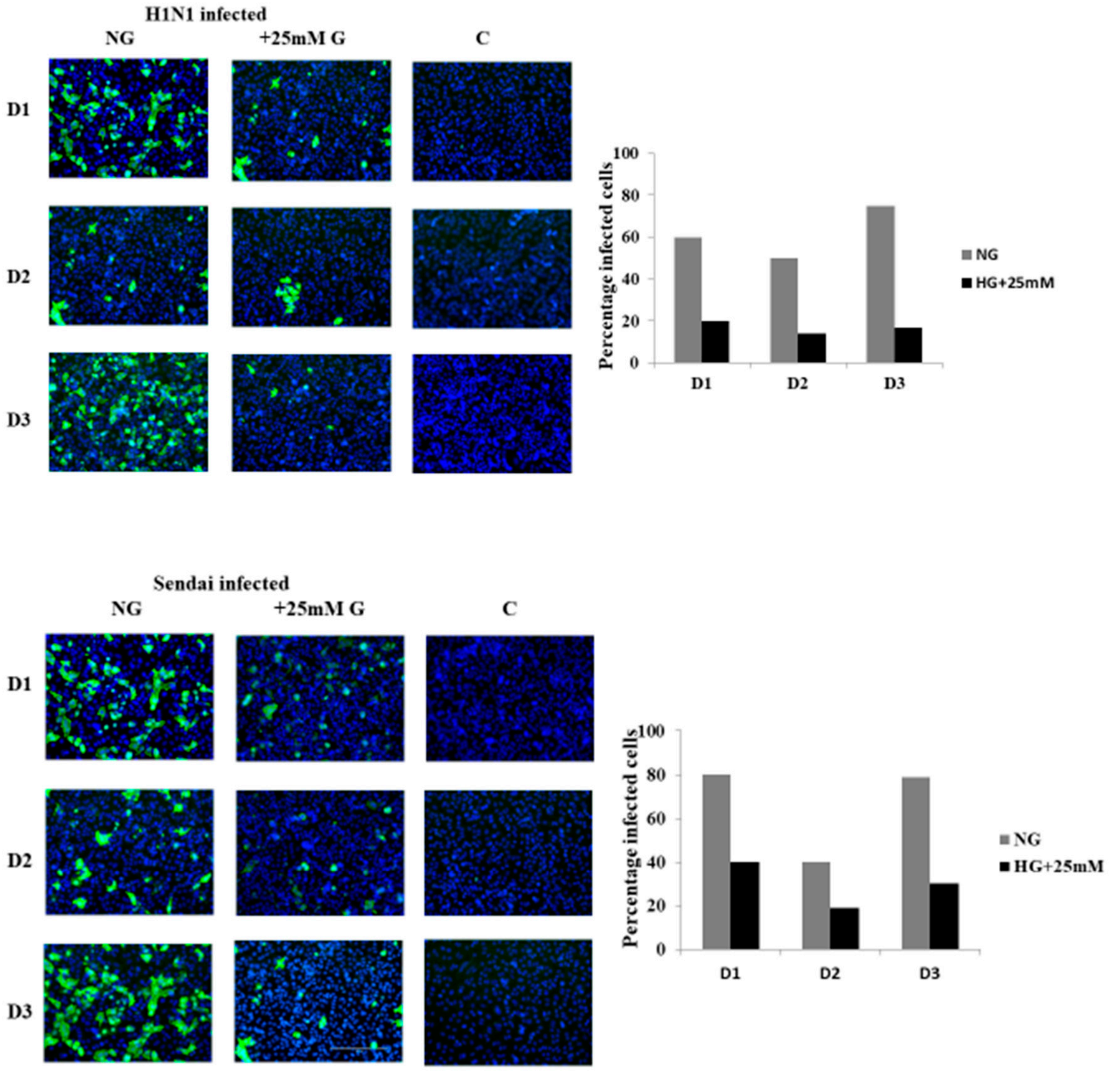

Figure S1. Determination of influenza and Sendai virus replication in normal and +25mM glucose concentrations in primary human macrophages by immunofluorescence (IF) staining.

2- Lactate concentration (nmol/μl) in culture media primary human macrophages infected with H1N1 or Sendai virus (MOI 10) at 8-12h PI in NG and +25mM G

Results show that in infected cells lactate concentration significantly increased in +25mM G compared to the control NG group 8-12h PI in primary cultured human macrophages.

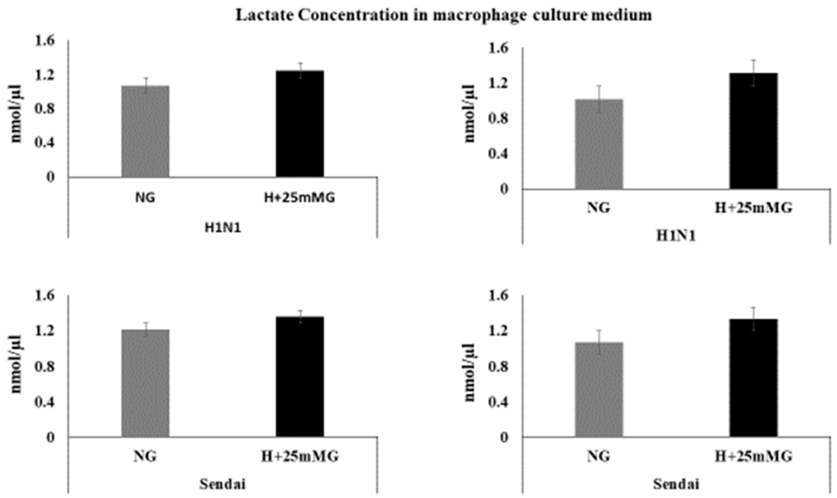

Figure S2 Lactate concentration (nmol/μl) in culture media primary human macrophages infected with H1N1 or Sendai virus (MOI 10) at 8-12h PI in NG and +25mM G

Material and methods

Culture and infection of primary human macrophages

Monocytes were isolated from buffy coat blood products from healthy blood donors, and macrophages were differentiated from monocytes as shown below. Buffy coat preparations were purchased from the Finnish Red Cross Blood Service, Helsinki, Finland. In brief, peripheral blood mononuclear cells were purified from 3 buffy coat preparations using Ficoll density gradient followed by allowing mononuclear cells to adhere to plates (1×10^6 cells per mL; Falcon; Becton Dickinson, Franklin Lakes, NJ, USA) for 1 h in RPMI 1640 medium (Sigma-Aldrich, St. Louis, MO, USA) at +37 °C. Macrophage serum free culture medium (Marcophage-SFM, Thermo Fisher Scientific, Waltham, MA, USA) was supplemented with penicillin/streptomycin, 20 mM HEPES, and 2 mM l-glutamine. Non-adherent cells were removed by washing with cold phosphate-buffered saline (PBS). The adherent monocytes were differentiated into macrophages by growing them in macrophage-SFM adjusted to levels of normal (~17.5 mM) and high (+25 mM resulting in 42.5 mM) D-glucose (Sigma-Aldrich, USA) before being supplemented with penicillin/streptomycin, 20 mM HEPES, 2 mM l-glutamine, and human recombinant granulocyte macrophage colony-stimulating factor (GM-CSF; 10 ng/mL; Nordic Biosite, Täby, Sweden). The cells were

differentiated into macrophages for 7 days with a change of fresh culture medium every 2 days and monitoring of differentiated macrophages via light microscopy. Infection with either H1N1 or Sendai virus was done as previously described [16, 36]. The infectivity titers of the stock viruses in cells were 6×10^7 PFU/ml and 1×10^9 PFU/ml for H1N1 strain and Sendai virus, respectively. Viruses were used at an adjusted multiplicity of infections (MOI 20). Cells were subjected to immunofluorescence microscopy (IF) or culture supernatants and lysates were collected at the different times and used for RNA, enzymatic and other analyses or stored at -80°C.
